# Mung Bean nuclease mapping of RNAs 3' end

**DOI:** 10.1186/1742-4933-6-6

**Published:** 2009-05-21

**Authors:** Daniele Bellavia, Giorgia Sisino, Giorgio L Papadopoulos, Giusi I Forte, Rainer Barbieri

**Affiliations:** 1 Dipartimento di Biologia Cellulare e dello Sviluppo – Università di Palermo – V.le delle Scienze, Edificio 16, 90128 Palermo, Italy; 2 Patologia Clinica, Dipartimento di Biopatologia e Metodologie Biomediche – Università di Palermo, Palermo, Italy

## Abstract

A method is described that allows an accurate mapping of 3' ends of RNAs. In this method a labeled DNA probe, containing the presumed 3' end of the RNA under analysis is allowed to anneals to the RNA itself. Mung-bean nuclease is then used to digest single strands of both RNA and DNA. Electrophoretic fractionation of "protected" undigested, labeled DNA is than performed using a sequence reaction of a known DNA as length marker. This procedure was applied to the analysis of both a polyA RNA (Interleukin 10 mRNA) and non polyA RNAs (sea urchin 18S and 26S rRNAs). This method might be potentially relevant for the evaluation of the role of posttrascriptional control of IL-10 in the pathogenesis of the immune and inflammatory mediated diseases associated to ageing. This might allow to develop new strategies to approach to the diagnosis and therapy of age related diseases.

## Findings

S1 mapping and primer extension are methods used to map the 5' end of an RNA [1]. Although the mapping of 3' ends of RNAs is often as important as the mapping of correspondent 5' ends (i.e. for the presence in the so-called 3' untranslated regions of sequence motifs linked to mRNA stability and/or to map 3'-ends of *non *polyA RNAs) there is, however, no standard procedure for mapping the 3' end of RNAs.

We developed a simple, reliable method to map the 3' ends of both poly-A and *non *poly-A RNAs.

**Figure 1:**
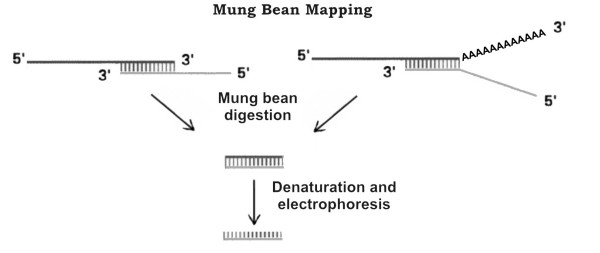
**Schematic description of Mung Bean mapping method**.

This method is reminiscent of S1 mapping [1], and makes use of a labelled DNA probe complementary to the 3'-end of the RNA, which contains the presumed 3' end of the RNA itself. After the probe was annealed to its target, Mung Bean nuclease is used to digest single strands of both DNA and RNA (see Figure 1). Mung Bean nuclease [2] is in fact a single strand-specific nuclease which digests DNA or RNA with a higher specificity than S1 nuclease [1]. This avoid incomplete digestion of single strand components that often occurs when S1 nuclease is used.

After denaturation, the undigested single strand DNA complementary to the 3' end of RNA is electrophoretically fractionated side by side with a sequence reaction of a known DNA used as a marker, which allows the measurement of the length of the undigested, labelled DNA probe. The procedure is reassumed in Figure 1.

We have recently demonstrated (manuscript submitted) in human white blood cells cultured in the presence of LPS, the existence of two interleukin-10 (IL-10) mRNAs, which differ in the length of the 5' UTR regions. To verify if the 3' ends of these mRNA also differ in their respective lengths we used the procedure reported above.

Moreover, to demonstrate that our procedure works well also with *non *poly-A RNAs, we mapped the 3' ends of the sea urchin *Paracentrotus lividus *18S and 26s mature ribosomal RNAs (rRNAs).

For this purpose we used labelled DNA clones of known sequence [EMBL AM981272, EMBL NC000001] [3] as it is certain that they contain the 3' ends of the aforementioned RNAs, obtained by specific PCR [1] on correspondent genomic DNAs in the presence of a labelled dNTP.

**Figure 2:**
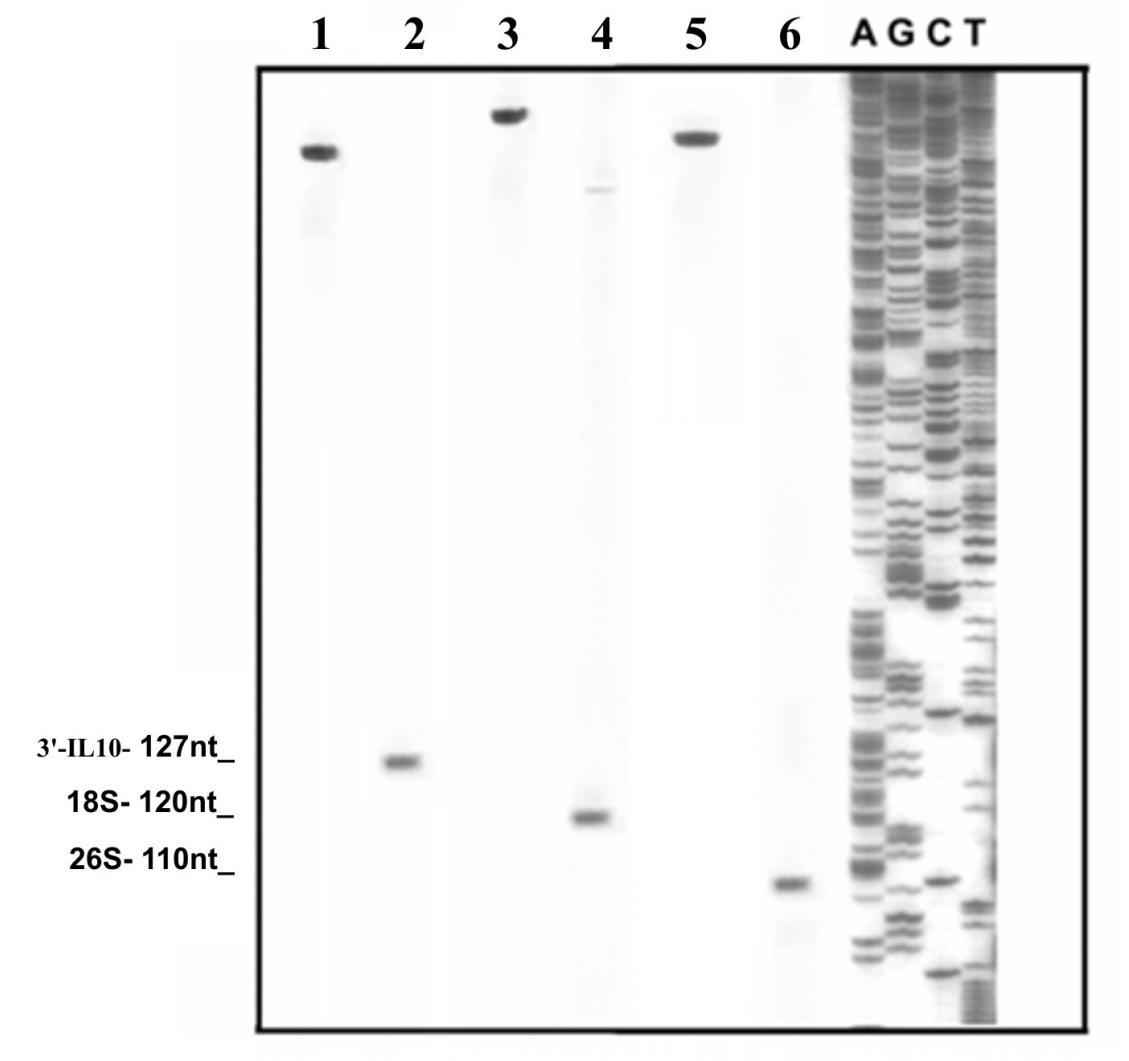
**Electrophoretic fractionation of the "protected" labelled probes obtained using our procedure in the mapping of human IL10 RNA (lane 2) and *P. lividus *18S and 26S rRNA (lanes 4 and 6, respectively) 3' ends, with respect to a known DNA sequence (in the right)**. In lanes 1, 3 and 5 the corresponding undigested probes are shown.

Aliquots (10 μg each) of total RNA extracted as indicated elsewhere [3] from both *P. lividus *unfertilized eggs and human blood white cells cultured in the presence of LPS, were incubated at 65°C for 5 minutes to denature all the possible secondary structure in the RNA. The denatured RNAs were then used to anneal, at 65°C for 5 minutes, with PCR amplified (Taq DNA polymerase native, Invitrogen™, Canada) labelled DNA fragments, pre-incubated at 95°C for 3 minutes, which we know to contain the 3' ends of correspondent RNAs [EMBL AM981272, EMBL NC000001] [3]. Incubation of the annealed molecules was carried out for 10 minutes at 37°C in the presence of 30 U of Mung Bean nuclease (Amersham™, Germany). After heat denaturation of the digested samples, the resulting DNA fragments were analysed by electrophoresis on 10% polyacrilamide gel [4] side by side with a sequence reaction (CycleReader™ DNA sequencing kit, Fermentas, Lithuania, according to manufacturer's protocol) of a DNA of known sequence as a length marker. Figure 2 shows the result of this experiment.

In lanes 2, 4 and 6, bands of 127 nucleotides (nt), 120 nt and 110 nt, corresponding to the IL-10, 18S and 26S RNA digestions, respectively, indicates the length of the "protected" regions, permitting an accurate mapping of respective 3' ends by comparison with the sequence reaction. In lanes 1, 3 and 5, IL-10, 18S and 26S undigested probes, respectively, were fractionated.

The accuracy and sensibility of our system is also demonstrated by the presence of a faint band in lane 4, which represents the 3' end of a 21S rRNA, precursor of the mature 18S rRNA. The presence of low amounts of this precursor was previously demonstrated [3] in *P. lividus *unfertilized eggs. If a "long run" of the electrophoresis shown in Figure 2 is performed, it would be easy to map also the 3' end of this pre-rRNA.

Taking into account the well known role of IL-10 in longevity and in age-related diseases [5-7], we have described a method that might be potentially relevant for the evaluation of the role of posttrascriptional control of IL-10 in the pathogenesis of the immune and inflammatory mediated diseases associated to ageing. This might allow to develop new strategies to approach to the diagnosis and therapy of age related diseases.

## Abbreviations

rRNA: ribosomal RNA; mRNA: messenger RNA; IL10: interleukin 10; dNTP: deoxyribonucleotides; nt: nucleotides.

## Competing interests

The authors declare that they have no competing interests.

## Authors' contributions

DB carried out both the molecular and the electrophoretic procedures and helped to draft the manuscript. GS participated to preparation of the materials used in the experiments and participated to all the experiments described in the text. GLP participated to all the experiments described in the text. GIF participated to all the experiments described in the text. RB senior author; conceived of the study, and participated in its design and coordination and wrote the manuscript.
